# Introduction of salvage prostatectomy in Denmark: the initial experience

**DOI:** 10.1186/s13104-022-06076-2

**Published:** 2022-05-21

**Authors:** Mike Allan Mortensen, Charlotte Aaberg Poulsen, Göran Ahlgren, Kirsten Madsen, Mads Hvid Poulsen

**Affiliations:** 1 Department of Urology, Odense University Hospital, Odense, Denmark; 2 Peritus Clinic, Lund, Sweden; 3 Department of Pathology, Odense University Hospital, Odense, Denmark; 4 Institute of Molecular Medicine, University of Southern Denmark, Odense, Denmark; 5 Department of Clinical Research, University of Southern Denmark, Odense, Denmark

**Keywords:** Prostatic neoplasms, Recurrent disease, Prostatectomy, Salvage treatment

## Abstract

**Objective:**

To introduce salvage prostatectomy in Denmark. Prior to this, no national curative treatment for recurrent prostate cancer following radiation therapy existed in Denmark. This pilot study represent our initial experiences and the feasibility of performing salvage robot-assisted radical prostatectomy for true local, high-risk recurrence after initial therapy with external beam radiation for high-risk prostate cancer.

**Results:**

Five patients underwent sRARP between April 2020 and July 2021. All patients were discharged within 48 h and no major complications were observed within 3 months. All patients had unmeasurable PSA (< 0.1 ng/ml) at follow-up 6 months after surgery. One patient with longer follow-up than 6 months experienced biochemical recurrence. At 3-months follow-up all patients reported considerable incontinence, at 6-month follow-up, pad usage decreased to 1 or 2 pads daily. Based on our initial results, the idea to introduce sRARP as a nationwide option remains and further patients will be included to establish the true role of sRARP in patients with recurrence after primary radiotherapy for PCa.

## Introduction

Despite continuous efforts to improve outcome of primary treatment for Prostate cancer (PCa), a considerable number of patients treated with radiotherapy will experience biochemical recurrence (BCR) defined as a prostate specific antigen (PSA) of 2 ng/mL + nadir. The risk of recurrence ranges from 15 to 25% for low-risk disease and 30–60% for patients with high-risk disease [1–4].

The natural history of PCa is highly heterogenous with a disease spectrum ranging from highly aggressive to an almost indolent disease. This is also the case for recurrent disease after definitive treatment with some patients experiencing very indolent disease that may never develop to metastatic disease. Historically, BCR after radiation therapy has been shown to be a poor surrogate for overall survival as well as cancer-specific mortality with older studies suggesting that BCR precedes clinical metastases by as much as 7 years [5]. Contemporary studies have contrarily suggested that BCR after treatment for high-risk disease significantly impacts overall survival as well as cancer-specific survival [6]. With the introduction of prostate-specific membrane antigen (PSMA) positron emission tomography/computed tomography (PET/CT), early detection of local recurrence has been possible, creating a window of opportunity for salvage treatment [7].

Salvage treatment of locally recurrent prostate cancer remains controversial due to the rather high rates of urinary and gastrointestinal toxicity associated with any salvage therapy [8]. According to recent European Association of Urology guidelines, salvage robotic assisted radical prostatectomy (sRARP) could be considered in healthy males with long life expectancy and favorable disease characteristics at primary diagnosis [9].

In Denmark, we estimate that 100 patients every year have recurrent prostate cancer following primary radiotherapy of prostate cancer. Up until now, no national strategy for curative treatment for recurrent prostate cancer following radiation therapy have existed in Denmark. This case analysis is the first step towards systematically introducing (sRARP) in Denmark with the purpose of describing safety and morbidity.

In the following we report our pilot study of the first five cases of sRARP from our institution performed on patients with local recurrence after primary external beam radiation. The study is based on the Danish Prostate Cancer Group (DaProCa) and is supported by the Danish Health Authorities.

## Main text

### Patient selection

From April 2020 to July 2021, 18 patients were screened for eligibility to undergo sRARP. All patients screened underwent 18F-PSMA-PET/CT, prostate MRI as well as 6- to 12-core transrectal prostate biopsies as recommended by the European Association of Urology guidelines [9]. MRI was solely used to determine local stage including detection of eventual rectal, bladder or pelvic muscle involvement. MRI was not used for image guided biopsy.

Inclusion criteria for sRARP were BCR following primary external beam radiation and an expected life expectancy of 10 years or more. Patients with more unfavorable disease characteristics than suggested by the European guidelines was acceptable [9]. Exclusion criteria were metastatic disease based on PSMA-PET/CT or inoperability due to extensive local disease based on MRI.

Following multidisciplinary team conference, five patients were considered eligible for sRARP. Exclusion of the remaining 13 patients was based on metastatic disease (n = 7), comorbidity (n = 3), extensive local disease (n = 2) and patient preference (n = 1) Patients were all pretreated with androgen deprivation for 1–3 months prior to surgery. Androgen deprivation was in all cases administered after both PET/CT and MRI. No androgen deprivation was administered after surgery.

### Surgical procedure and post-operative management

All included patients underwent sRARP with a standard transperitoneal anterior approach with no nerve sparring. No drain was placed in any patient. Nodal dissection was not performed as lymph nodes previously had received radiation treatment and in the absence of PET-positive nodes. All procedures were performed one surgeon (MHP) with extensive experience in RARP (over 400 RARP-cases) and under supervision of a surgeon (GA) with extensive experience in sRARP (1000 RARP-cases and over 30 salvage cases) from his own institution. The Department of Urology at Odense University Hospital annually performs around 250 RARP cases.

All patients were administered 1.5 g of cefuroxime prior to surgery. The anesthetic protocol did not differ from the one used for standard RARP.

All patients were discharged with an indwelling transurethral catheter planned for removal after cystography performed 14 days post-operatively. The longer period with indwelling catheter in patients undergoing sRARP compared to normal procedure with catheter removal after 8–10 days with no cystography in patients undergoing RARP, was based on experience and recommendations from the supervising surgeon. All patients were evaluated by an experienced physiotherapist 2–4 weeks after surgery with instruction in pelvic floor exercises (Kegel exercises). Long-term functional outcomes were collected via patient reported outcome measures.

### Statistics

Patient demographics were analysed using descriptive statistics. All analyses were performed using STATA/IC 15.1 (StataCorp, College Station, Texas, USA).

### Results

Five patients underwent sRARP between April 2020 and July 2021. All patients had recurrent disease after external beam radiation to the prostate with 78 Gy in 39 fractions. All patients received 3 years of androgen deprivation therapy in conjunction with radiotherapy. The time between radiation therapy and sRARP varied widely ranging from 40 to 128 months (median 65 months). All patients had primary treatment for high risk PCa with clinically advanced disease (cT3a or cT3b) and high Gleason score (Gleason score 8 or above in 4 cases). All patients had biopsy proven recurrent prostate cancer. Three patients had extensive recurrent disease based on prostate biopsy with the majority of biopsy cores being infiltrated with PCa.

The median age at the time of sRARP was 71 years ranging from 67 to 74 years. Median PSA prior to sRARP was 3.8 ng/mL (range 2.2–4.0 ng/mL). Summarized data of the included patients can be seen in Table 1.

**Table 1:** Summarized data of the included patients at diagnosis and at time of sRARP

Patient no.	Status at diagnosis				Status at sRARP						
	PSA (ng/mL)	Clinical stage	No. of positive cores	Gleason score	PSA (ng/mL)	Pathological stage	No. of positive cores	Estimated extension (%)	Gleason score	Surgical margins	Androgen deprivation
1	8.8	cT3b	12/12	3 + 4	2.2	pT3a	5/6	40	4 + 3	Negative	Bicalutamide
2	52.0	cT3b	10/11	4 + 5	3.8	pT3b	1/6	30	4 + 5	Positive	Degarelix
3	22.0	cT3b	7/10	4 + 5	2.8	pT2a	4/12	10	4 + 5	Negative	Triptorelin
4	61.0	cT3a	12/12	4 + 5	4.0	pT3b	12/12	75	5 + 4	Positive	Triptorelin
5	7.8	cT3a	7/7	4 + 4	3.9	pT2c	12/12	40	4 + 3	Positive	Triptorelin

Operative time (defined as time in surgical console) ranged from 160 to 250 min. No major intra-operative complications were observed. Median blood loss was 120 ml (range: 50–150 ml).

Three patients suffered from minor post-operative complications according to the Clavien-Dindo grading [10]. One patient experienced anastomotic leakage resulting in a prolonged period of 30 days with indwelling transurethral catheter (Clavien-Dindo grade II). The leakage was observed on routinely performed cystogram.

All patients were discharged within 2 days with no need for readmission in any case. Two patients were treated for suspected urinary tract infection with oral antibiotics (Clavien-Dindo grade II).

**Fig. 1:**
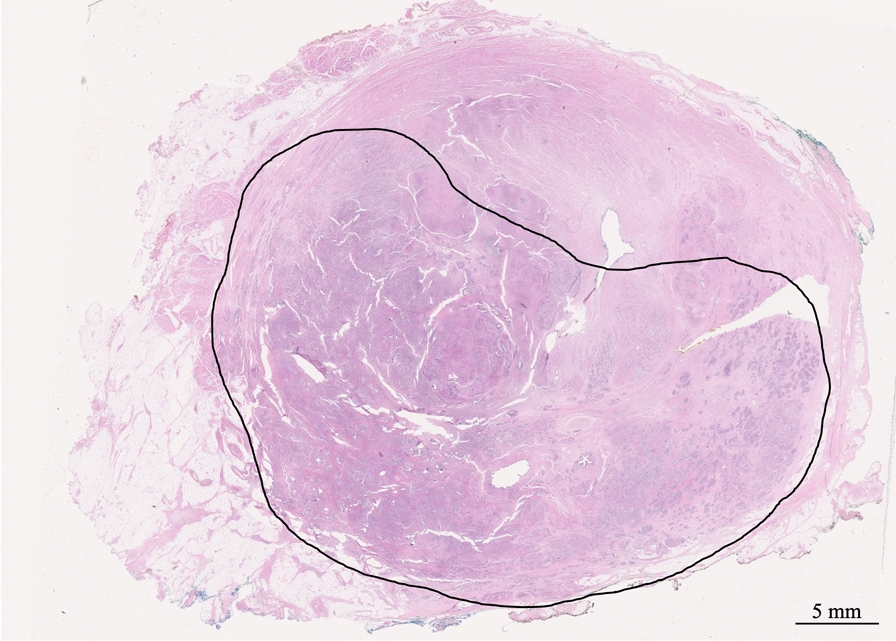
Whole-mount section of the prostate showing massive tumor infiltration. Gleason score 9. Hematoxylin and eosin stain

**Fig. 2:**
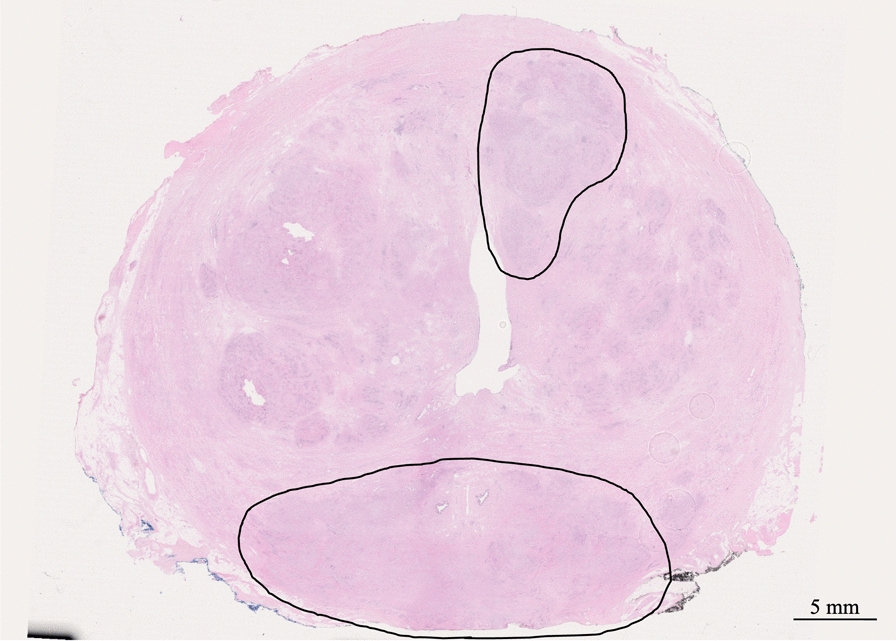
Whole-mount section of the prostate showing multi-focal tumor infiltration. Gleason score 9. Hematoxylin and eosin stain

Post-operative histological examination revealed significant disease in all patients with Gleason grades similar to the ones observed at preoperative biopsies and estimated extension within the prostate ranging from 10 to 75% (Fig. 1). In addition, the recurrent tumor was also found to be multi-focal in most patients (n = 4) (Fig. 2). Three patients had positive surgical margins of 1.5, 5 and 7 mm, respectively.

All patients had unmeasurable low PSA at 3- and 6-months follow-up. The median follow-up was 13 months (range 7–20 months). One patient experienced biochemical recurrence during follow-up with a measurable PSA 8 months after surgery.

None of the included patients reported incontinence, defined as no pad usage prior to sRARP. Three months after surgery all five patients reported considerable incontinence using 3 or more pads daily. At 6-month follow-up, pad usage decreased to 1 or 2 pads daily. For the patients with longer follow-up the incontinence has continued to improve. All included patients described severe sexual dysfunction prior to sRARP. Based on this sexual dysfunction was not evaluated after sRARP.

### Discussion

In this case study we report on the first experience performing sRARP at a single high-volume center. Based on five cases, sRARP appears to be a safe procedure when performed in highly selected patients. None of the operated patients in our study suffered severe complications although incontinence rates after sRARP seems high compared to patients after RARP where most patients observe considerable improvement in continence in the first months after surgery. The results from our study adds to the already existing knowledge on sRARP. sRARP was first introduced in the late 00’s [11, 12]. Before this, salvage surgery had been performed sporadically as open retropubic surgery.

All patients in our study had unmeasurable PSA 6 months after surgery suggesting excellent, immediate post-operative disease control. Early post-operative PSA measurements e.g., after 1–3 months, could very well be impacted by the androgen deprivation all patients received prior to sRARP in our study. PSA measurements performed at 6-months follow-up should not be influenced by pre-operative androgen deprivation and reflects disease control achieved by the surgical procedure alone.

One patient had re-recurrent disease 8 months after surgery with a PSA above 0.2 ng/mL. Based on data from previous series on salvage prostatectomy it is to be expected that a large proportion of patients operated will have biochemical and eventual clinical failure despite unmeasurable PSA directly after surgery. A meta-analysis from 2012 suggest that 18–53% of patients will have a measurable PSA within 5 years of surgery where [13]. These data are further supported by more contemporary studies with a recent multi-center study on sRARP suggesting a 5-year biochemical recurrence-free survival of 56.7% [14].

The major concern after sRARP has primarily been a high risk of post-surgical incontinence as well as high complication rates reported in early surgical series [15–17]. This is also seen in our study with considerable incontinence at 6 months reported by all patients. Despite the short follow-up in our study, incontinence seemed to improve rather rapidly although none of the patients regained complete continence during follow-up. However, none of the patients were considered to need evaluation for artificial sphincter surgery. This illustrates that preserving the continence mechanisms is a challenge and require that sRARP is performed in high-volume centers only [18].

Several options for salvage therapy after initial radiotherapy has been suggested including but not limited to sRARP, High Intensity Focus Ultrasound (HIFU), cryotherapy and re-radiation. A recent metanalysis showed comparable outcome but different side effects favoring re-radiation [8]. Series on salvage therapy are almost exclusively retrospective case-series with no randomized studies comparing given modalities.

Based on the multifocal nature of the recurrence seen in the majority of our patient, treatment targeting the whole gland might be advisable for at least a subset of patients whereas patients with unifocal recurrence might be better treated with focal therapy such as HIFU. Whether this is true and whether it is even possible to make this distinction before salvage treatment is up to question. Other questions that still needs attention is to whether given salvage modalities performs differently depending on the primary treatment that the patients have undergone.

It is generally accepted that sRARP should only be offered to highly selected patient with long life expectancy and localized disease only. With the advent of the PSMA-PET/CT and prostate MRI it is now possible to perform considerably better staging of recurrent disease making it possible to better identify patients with true local, high-risk recurrence [9, 19].

In this pilot study, sRARP appears safe in highly selected patients with no major per- or postoperative complications. In addition, sRARP appears to offer good tumor control, but the effect on urinary continence needs to be considered. Similar results have been reported in other series.

Based on the results of this pilot series, the idea to introduce sRARP as a nationwide option in Denmark remains and we will continue to include and evaluate patients to establish the true role of sRARP in patients with recurrence after primary radiotherapy for PCa. The patients will be followed closely for a period of five years to continuously evaluate safety and the effect on the cancer specific survival.

## Limitations

This study is limited by its small sample size. The rather short follow-up does not allow for any conclusions on long-term outcome.

## Data Availability

The data analysed in the present study may be obtained from the corresponding author upon request, subject to current restrictions pertaining, among others, to the research participants’ privacy.

## Abbreviations

BCR: Biochemical recurrence
MRI: Magnetic resonance imaging
PCa: Prostate cancer
PET/CT: Positron emission tomography/computed tomography
PSA: Prostate-specific antigen
PSMA: Prostate-specific membrane antigen
sRARP: Salvage robot-assisted radical prostatectomy
